# Approaching the post-antibiotic era: phage therapy current practices and future horizons

**DOI:** 10.1128/mbio.03254-25

**Published:** 2026-07-30

**Authors:** Madison E. Stellfox, Justin Massey, Kailey Hughes Kramer, Ryan K. Shields, Kenneth L. Urish, Ghady Haidar, Daria Van Tyne

**Affiliations:** 1 Division of Infectious Diseases, Department of Medicine, University of Pittsburgh 6614, Pittsburgh, Pennsylvania, USA; 2 Pittsburgh Phage Program, University of Pittsburgh 6614, Pittsburgh, Pennsylvania, USA; 3 Center for Evolutionary Biology and Medicine, University of Pittsburgh 6614, Pittsburgh, Pennsylvania, USA; 4 Division of Pediatric Infectious Diseases, Department of Pediatrics, University of Pittsburgh 6614, Pittsburgh, Pennsylvania, USA; 5 Bethel Musculoskeletal Research Center, Department of Orthopaedic Surgery, University of Pittsburgh 6614, Pittsburgh, Pennsylvania, USA; 6 Department of Bioengineering, University of Pittsburgh 6614, Pittsburgh, Pennsylvania, USA; 7 Clinical and Translational Science Institute, University of Pittsburgh 6614, Pittsburgh, Pennsylvania, USA; 8 Arthritis and Arthroplasty Design Group, The Bone and Joint Center, Magee Women’s Hospital of the University of Pittsburgh Medical Center, Pittsburgh, Pennsylvania, USA; Georgia Institute of Technology, Atlanta, Georgia, USA

**Keywords:** phage therapy, antibiotic resistance, bacterial infections

## Abstract

Bacteriophages (phages) are the viral predators of bacteria. Their ability to infect and kill bacterial strains has been harnessed for use in clinical practice for more than a century, but their development stalled for decades in many regions. Now, in the face of increasingly complex bacterial infections that fail conventional therapeutic options, and the growing global threat of antibiotic resistance, interest in phage therapy has resurged. Data evaluating the impact of phage therapy via clinical trials are steadily increasing, but remain sparse. Yet, an ever-growing number of case reports and case series highlights the potential of phage therapy to make a positive impact in the battle against antimicrobial resistance and difficult-to-treat bacterial infections. In this review, we summarize the available data describing the clinical utility of phage therapy, highlight infectious indications where phage therapy shows strong potential, and call attention to disease states where the benefits of phage therapy, as it is practiced today, are less certain. Furthermore, we note areas where there is a crucial need for additional research and propose how scientists and clinicians can work together to design and carry out the preclinical and clinical studies that will take phage therapy into its next century.

## INTRODUCTION

## A BRIEF PHAGE PRIMER

Phages are the naturally occurring viruses of bacteria (see Table 1 for key definitions). They have co-evolved with their bacterial prey across billions of years, resulting in highly specific phage-host interactions, with most phages able to infect only a few strains of a particular bacterial species. Phages infect their prey through two major replication cycles—lysogeny and lysis (1). Both cycles require a phage to encounter a bacterial host cell, bind to its surface, and then inject the bacterium with its viral genetic material. Lysogeny results in a phage’s genetic material being integrated and maintained alongside the bacterial chromosome in a largely quiescent state. During a lytic infection, the phage hijacks the bacterial host cellular machinery to synthesize and assemble viral progeny, eventually killing its bacterial host via lysis to release new infectious viral particles. Many phages employ both lifecycles, but some phage-host pairings are obligately lytic, meaning that the phage is unable to undergo lysogeny and is always lethal upon infecting susceptible bacterial hosts. Such obligately lytic phages are currently preferred for phage therapy, as they reduce the chance of transferring potentially problematic genetic material between bacteria via phage infection.

**TABLE 1:** Definitions of common phage therapy-related terms and principles

Term	Definition
Bacteriophage (phage)	Natural viral predator of bacteria
Lytic cycle	A viral replication cycle where an infecting phage’s genetic material uses the bacterial host’s protein synthesis machinery to produce viral progeny, killing (or lysing) the bacterium in the process
Obligately lytic	Phages that can only replicate via the lytic cycle
Lysogeny	A viral replication cycle in which a phage’s genome is stably maintained alongside the bacterial host’s chromosome until a signal inspires phage genome excision and entrance into a lytic replication cycle
Burst size	The number of phage progeny that can arise from a single phage-infected bacterial cell
Plaque	Clearing of a bacterial lawn caused by the lytic activity of a phage infection
Phage titer	A measure of the lytic activity of a phage preparation, usually expressed in plaque-forming units per milliliter (PFU/mL)
Susceptibility assay	An assay that measures whether a specific bacterial strain is able to be infected and lysed by a particular phage preparation
Host range	How broadly a phage preparation can infect and lyse a given bacterial species: Broad host range—lyses many different types of strains, and possibly more than one species Narrow host range—lyses only a select subset of strains of a particular species
Multiplicity of infection (MOI)	The estimated number of phage particles per bacterial cell in a given infection scenario. Calculated by dividing the number of plaque-forming units by the number of colony-forming units
Reticuloendothelial system	Phagocytic cells and their associated organs (mainly the liver and spleen) thought to be responsible for the majority of phage clearance from the blood following intravenous administration
Compassionate use	Clinical use of medical interventions not yet approved by the FDA, generally limited to patients with severe or difficult-to-treat conditions. Treatment must be approved by both the FDA and a local institutional review board

The ability of phages to prey upon and kill their bacterial hosts has been known since the early 1900s, and shortly after their discovery, phages were utilized clinically as antimicrobial therapy (2). However, this was in the era before DNA was known to be the heritable molecule and prior to the invention of electron microscopy techniques that could visualize viral particles. As such, rigor and reproducibility of both laboratory and clinical findings with phages were difficult to achieve. The discovery of phages also overlapped with the advent of the antibiotic era, starting with the synthesis of salvarsan and the discoveries of sulfonamides and penicillin in the following decade (3). Coupled with geopolitical tensions following World War II, when phage research was concentrated in Eastern Europe and the former Soviet Union, the study of phages and their clinical use diminished in Western medicine (4).

However, clinical interest in phage therapy has rebounded during the last two decades for several reasons. First, rising rates of antimicrobial resistance coupled with a slow antibiotic development pipeline are a significant concern, with a widely cited estimate that 10 million deaths per year could be attributable to antimicrobial resistance by 2050 (5). Next, a growing proportion of the human population in the United States is immunocompromised, a trend that is likely recapitulated worldwide (6). Finally, human health has been improved by the increasing use of indwelling medical devices, such as prosthetic joints and heart valves, artificial vascular grafts, and implantable cardiac electronic devices like pacemakers and left-ventricular assist devices (LVADs) (7–11). Yet indwelling medical devices can serve as niduses for bacterial infections, which can become chronic and difficult to treat when an infected device cannot be removed from the body. As such, a growing population is at risk for the development of chronic bacterial infections, and we need adjunctive therapies that can work alongside traditional antibiotics to manage these challenging conditions. Phages could fill this gap and be a critical addition to the antimicrobial “toolbox,” but how these viral predators can be best deployed clinically remains unclear.

## A NARRATIVE REVIEW OF CURRENT PRACTICES IN PHAGE THERAPY

In the United States and in most other countries, phage therapy currently remains an experimental treatment, largely due to a lack of large, randomized controlled clinical trials (RCTs). Before the early 2010s, only a handful of clinical trials using phages were registered with the ClinicalTrials.gov online database, let alone published in peer-reviewed journals. In the past two decades, however, both basic science and clinical research focused on phage therapy have increased dramatically. Since 2010, more than 50 interventional trials (excluding repositories and single-patient investigational new drug protocols) have been registered, and more than two-thirds of these have study initiation dates post-2020.

This increase in phage-related trials is also reflected in the published literature. Many published clinical trials are early-phase, and the distinction between these and case series can be challenging to define. For the purposes of this narrative review, we defined a clinical trial as a study that recruited patients to receive a defined phage therapeutic product and followed patient outcomes via a pre-specified monitoring plan (Table S1). Of the more than 25 articles published in PubMed and EMBASE (written in English, with full text availability) that described such trials, more than three-fourths were published after 2010. Many of these studies were designed to evaluate the safety and tolerability of phage products in human patients, leaving only a handful that formally assessed phage therapeutic efficacy.

While fewer than 1,000 patients have been treated with phage therapy in published clinical trials, compared to other experimental antimicrobial treatments, phage therapy is relatively widely used. A review article published in 2021 by Uttyerbroek et al. estimated that more than 2,000 patients had been treated with phage therapy, and since then, the number of people who have received therapeutic phages has continued to climb (12). Perhaps unique in the field of medicine, ongoing interest and investment in the potential of phage therapy has not stemmed from RCTs, but rather has been compiled from the combined experience derived from hundreds of individual cases and small case series in which patients were treated with phage therapy via compassionate use or expanded access protocols (Table S2). While the results of these case reports and case series likely suffer from reporting bias and limited generalizability when reviewed individually, in aggregate and coupled with published RCTs, several general themes emerge.

First and foremost, phage therapy is remarkably safe in both pediatric and adult populations and when given via numerous routes of administration (12, 13). While the total number of patients treated with phage therapy in clinical trials remains small, minimal side effects have been documented. Additionally, through compassionate use and expanded access protocols, dozens of different phages at doses as high as 10^11^ virions have been administered, and similarly, few severe adverse events have been reported. Those that have occurred have predominantly been either transient inflammatory reactions or reversible elevations of liver transaminases (12, 14–17).

Second, and perhaps unsurprisingly, phage susceptibility testing confirming that the phages used for treatment are active against their target bacteria appears to increase the likelihood of phage therapy being efficacious. Phage-host interactions are highly specific, and phage susceptibility is determined by assessing whether a particular phage preparation can kill the target bacterial strain. This is traditionally performed by plaque assay using the double-layer agar method, where a lawn of target bacteria is suspended in gelled media and a phage preparation is dropped onto the lawn (18). If the bacterial strain is susceptible to the phage, the lawn under the drop will appear clear as compared to the turbidity of phage-unexposed regions, and this clearance zone is called a plaque. When drops of a serially diluted phage preparation are tested, this technique can also estimate the number of active phage particles in a preparation (i.e., phage titer). Alternative susceptibility tests usually measure a change in bacterial growth kinetics in liquid culture when the inoculum is exposed to a phage preparation, either by measuring optical density or bacterial cell metabolism (19). Many successful case reports describe personalized phage therapy protocols, with both the phage(s) and the route(s) of administration tailored to each patient’s specific infection. Conversely, several clinical trials that did not find evidence of efficacy either lacked the predetermination of phage susceptibility of infecting isolates or reported uncertainties about the phage dose applied (20–22). For treatment of burn wounds in particular, two clinical trials that did not use phage susceptibility of the infecting bacteria as an eligibility criterion failed to show that phage therapy was more effective than the comparator arms (21, 22). However, available case reports and case series of individual patients with burn wounds treated with phage therapy report much more favorable outcomes; in all cases, the infecting isolates were tested to confirm phage susceptibility prior to treatment (23–26).

Third, the greatest signals for potential efficacy appear to occur when the administered phage therapeutic is likely to achieve a high multiplicity of infection (MOI) at the infected body site. Phages that are given intravenously must find susceptible bacterial hosts present at the site of infection while being rapidly cleared from the blood via the reticuloendothelial system (27, 28). Infections that allow for direct, topical application of phage preparations bypass this elimination route and allow for an elevated MOI at the site of infection. When medically appropriate, phage therapy also appears to work best when it is coupled with surgical debridement, such as for infections of prosthetic material and indwelling medical devices. During surgery, it may be possible to apply phage preparations directly to the infected body site and/or foreign material. Surgical management also allows for mechanical debridement or exchange of material, lowering the bacterial burden at the site of infection and functionally increasing the local MOI.

## DISEASE STATES WHERE CLINICAL EVIDENCE SUGGESTS A POSSIBLE BENEFIT OF PHAGE THERAPY

**Fig 1:**
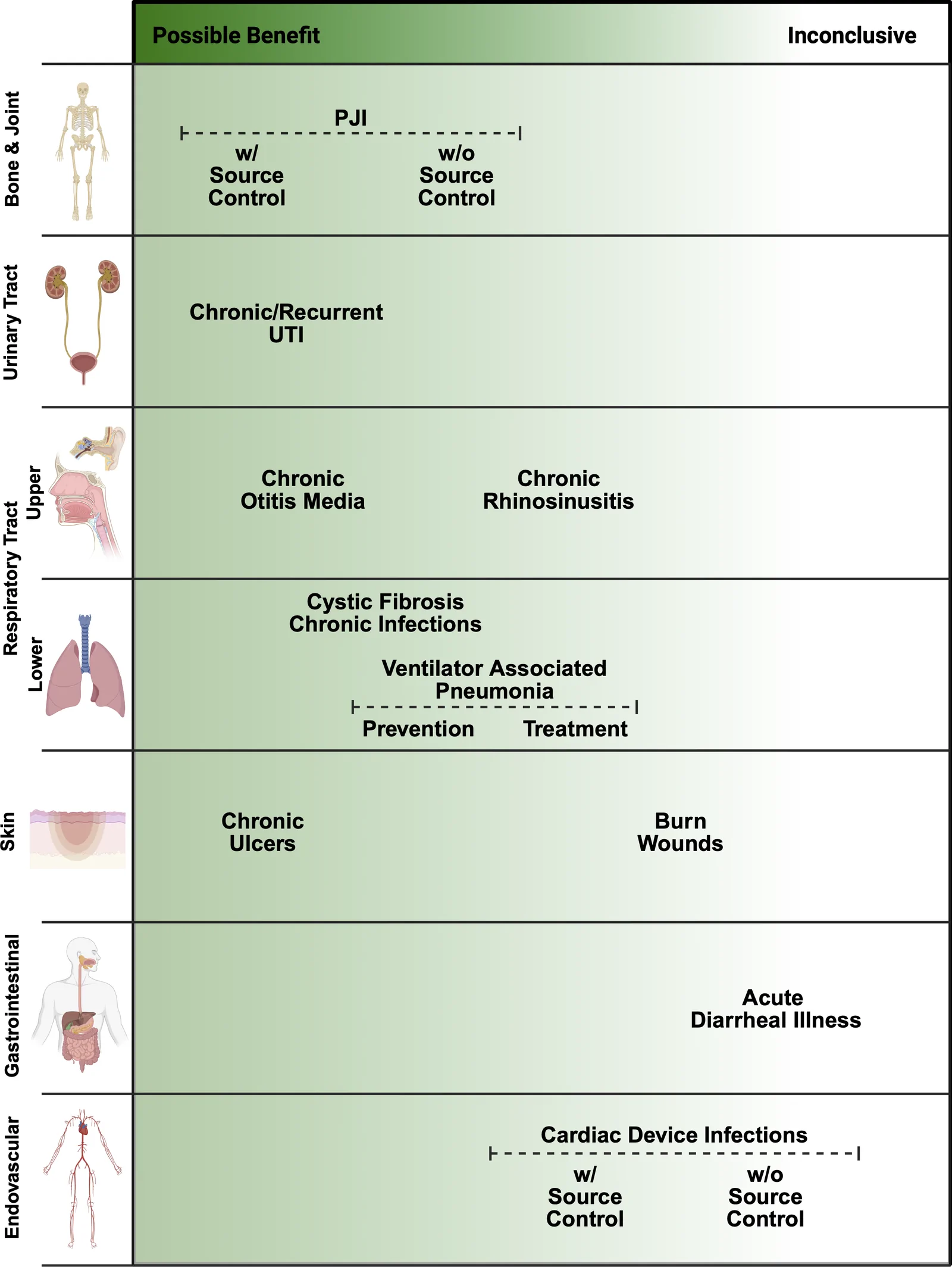
Infectious conditions that have been treated with phage therapy. Infections are separated by organ system and are placed along a gradient based on currently available published literature. Darker green shading means stronger evidence of a possible benefit of phage therapy, while lighter shading indicates that evidence of benefit is inconclusive.

Based on data from the small number of clinical trials and combined experience from global centers offering compassionate use phage therapy, evidence is emerging to suggest that some types of infection might be more responsive to phage therapy than others. These disease states largely adhere to the principles outlined in the preceding section, including that phage susceptibility testing before administration is feasible and that the route of application is likely to achieve high MOIs (Fig. 1).

### Bone and joint infections

Bone and joint infections are heterogeneous and include entities such as osteomyelitis (acute hematogenous, trauma-associated, and ulcer-associated), septic arthritis, and surgical site infections, each of which has been an indication for phage therapy (Table S2). Perhaps, the most clinically intriguing use of phage therapy for bone and joint infections is its role as a novel therapy for difficult-to-treat prosthetic joint infections (PJIs). Treatment failure of PJIs is associated with significant morbidity and mortality, and better interventions that can reduce amputations and repetitive surgical risks are necessary (29). PJIs are well-suited for phage therapy because they are usually chronic, smoldering infections, allowing sufficient time to determine the causative pathogen and perform phage susceptibility testing before initiation of therapy. Additionally, even in device retention and limb salvage protocols, management of PJIs often requires surgical debridement, which can help achieve high local MOIs. However, clinical trial data from studies of phage therapy for PJIs are incredibly sparse. At the time of writing, only one small, non-randomized clinical study compared the addition of phage therapy to standard-of-care (surgery and systemic antibiotics) for staphylococcal PJIs of the hip to historical controls (30). The authors reported a greater response to treatment and an 8-times lower rate of recurrence within a year of follow-up in the group treated with phage therapy. While the study was not intentionally designed to test a single phage preparation, all patients were treated with a single product (“Staphylococcal bacteriophage” produced by Microgen, Russia), which improves the comparative nature of this assessment. On the other hand, using historical controls has drawbacks. One potential confounder is the significant differences in the antibiotic treatments both groups received. All patients in the phage cohort received a total of 12–14 weeks of antibiotics, while patients in the historical control group were treated with a variety of antibiotic regimens, some of which were less than 12 weeks in duration (30).

While clinical trial data are currently limited, at least two trials have been registered that plan to assess phage therapy for *S. aureus* PJIs of the knee and hip in patients undergoing debridement and implant retention (DAIR) surgeries (NCT06605651, NCT05369104). In addition, several case series and reviews highlight that compassionate use phage therapy has consistently been shown to be of value in the management of bone and joint infections, particularly difficult-to-treat PJIs (31–35). A systematic review published in 2021 assessed phage treatment characteristics and outcomes of 51 bone and joint infections (29). Most patients underwent concomitant surgical debridement and received topical application of phage (with or without systemic phage administration), and phage was mostly used as an adjunctive therapy to systemic antibiotics. The rate of clinical success after phage therapy in these compassionate use cases appears to be quite high. Overall, it is estimated that hundreds of patients have received phage therapy for bone and joint infections through compassionate use and expanded access protocols, with general efficacy estimated to be greater than 70% (29, 31, 34, 35).

### Urinary tract infections

Urinary tract infections (UTIs) also appear to be well-targeted by phage therapy. In the case of chronic or recurrent cystitis, patients are usually well enough to allow for the development and manufacture of personalized phage therapeutics targeting the causative pathogen. Additionally, the anatomy of the urinary system allows for high MOI treatment of its structures. The kidneys are highly vascularized, which may allow for adequate phage exposure via intravenous administration, and phages have been detected in the kidneys after both oral and systemic administration in several animal models (36). The bladder is also easily accessible via minimally invasive procedures such as catheterization, during which a phage solution can be instilled, allowing for direct phage contact with the bladder mucosa.

As such, UTIs are one of the most frequently targeted disease processes in compassionate and expanded access phage therapy protocols, and successful treatment of recurrent cystitis (both in immunocompetent and renal transplant patients) as well as chronic bacterial prostatitis with phage preparations have been reported (Table S2). Local phage administration to the urinary system is very common in these reports, and most patients received phages by instillation into the bladder as part of their treatment regimen. Other routes were used as well, including instillations into the renal parenchyma (via percutaneous nephrostomy) (37), the vagina (38), or the rectum (39–42). Many patients also received systemic phage therapy by mouth or intravenously. While phage therapy alone was successful in at least one case (43), most patients received phages in combination with systemic antibiotics, and synergistic interactions between phages and even inactive antibiotics were reported for some targeted uropathogens (44, 45).

In addition to compassionate use cases, two clinical trials have examined the utility of phage therapy for UTIs (17, 46) and many more are currently enrolling. Leitner et al. published a trial in 2021 comparing an intravesicular phage cocktail to bladder irrigation or systemic antibiotics to eradicate uropathogens in males undergoing transurethral resection of the prostate (TURP) who had a symptomatic UTI with a pathogen susceptible to a Pyophage cocktail (46). The men who received intravesicular phage therapy experienced fewer adverse events than patients treated with antibiotics, but phage instillations were non-inferior to systemic antibiotics and non-superior to simple bladder irrigation, calling into question whether phage provided any real benefit. While this trial did include pre-phage testing to guarantee a phage-pathogen match, there were also several limitations. For example, the study was likely underpowered due to an incorrect estimate of anticipated success in both the phage and antibiotic treatment arms. There was also a high number of protocol deviations. Finally, the trial used a suprapubic catheter to perform both phage and bladder irrigations, and while that may be part of standard-of-care, placement is a surgical procedure and high healthcare utilization may have complicated trial adherence (46). Conversely, the initial results from the ELIMINATE trial deploying phage therapy in combination with trimethoprim-sulfamethoxazole for recurrent *E. coli* UTIs in adult females appear promising from an efficacy standpoint (17). Early stages of the study were challenged by adverse events (tachycardia and afebrile chills), possibly due to very high intravenous phage doses (10^11^ PFU/dose). A reduction in the intravenous phage dose or its administration rate appeared to largely resolve those issues. While not designed to formally assess efficacy, all patients who completed the trial had *E. coli* eradicated from their urine cultures and had resolution of their lower urinary tract symptoms, which lasted until the final follow-up at day 34 (17).

### Decolonization and treatment of the upper and lower respiratory tract

The upper and lower respiratory tracts appear to be well targeted by phage-mediated interventions, both as an adjunctive treatment for acute infection and as a pathogen decolonization strategy. In the upper respiratory tract and ear canal, direct application of phage to infected tissue is possible. Phages have also been successfully delivered to the lower respiratory tract via systemic administration and nebulization (36) although phage viability has been seen to vary between different nebulizer types (47). Treatment of pulmonary infections has been the most common use of compassionate use phage therapy to date (Table S2). Additionally, there have been multiple clinical trials assessing the use of phage therapy in both the upper and lower airways (Table S1).

With regard to the upper respiratory tract, three small clinical trials assessed the use of topical phage preparations to manage chronic infections of the sinuses and inner ear and found that phage treatment decreased the bacterial burden of the suspected pathogens (48–50). Two studies also included assessment of clinical efficacy. In the first study, phage treatment resulted in improved appearance and symptom scores in patients with chronic *Pseudomonas aeruginosa* otitis media (49). In the second study, a single-arm dose escalation trial, phage was used to treat chronic *Staphylococcus aureus*-mediated rhinosinusitis and resulted in a reduced bacterial load from endoscopically obtained sinus cultures in all patients, with 22% of patients (2/9) achieving microbiologic cure and clinical resolution (50).

Additional studies have been performed assessing the use of phage therapy for infections of the lower respiratory tract. Two clinical trials evaluated the use of phages targeting *S. aureus*, *P. aeruginosa,* and *Acinetobacter baumannii* to reduce the occurrence of hospital-acquired pneumonia in patients with severe respiratory conditions, and in both studies, phage reduced the occurrence of either ventilator-associated pneumonia (VAP) or severe secondary pulmonary infection (51, 52). Additionally, a phase 1 trial evaluated an inhaled phage cocktail as adjunctive therapy to systemic antibiotics for multidrug-resistant *Klebsiella* ventilator-associated pneumonia, allocating 21 patients into 3 equal arms that received inhaled active phages, inactive phages, or placebo for 14 days in addition to standard antibiotic therapy (53). While limited by non-randomization, small sample sizes, and highly heterogeneous isolates based on capsule type and baseline antimicrobial susceptibility, active phage therapy was associated with improved microbiologic eradication (6/7 patients) compared to both the inactive phage (0/7) and placebo (4/7) groups. Moreover, 3/7 patients who received active phage therapy were able to discontinue antibiotics prior to day 14, compared to only a single patient in both the inactive phage and placebo arms (53).

Phage therapy has also shown potential in the management of chronic, antibiotic-resistant infections in persons with cystic fibrosis (pwCF). A recent systematic review analyzed data collected from 51 pwCF who received phage therapy either via compassionate use or through a clinical trial (54). While outcomes varied by infecting pathogen, most participants had evidence of microbiologic response (68%) and improved lung function (74%) post-therapy, with few phage-related adverse events. Part of this analysis included the results from a recent case series where nine pwCF were treated with inhaled phage for refractory *P. aeruginosa* pulmonary infections and which showed a decrease in pathogen burden and improved lung function (55). Also included was a phase 1b/2a trial comparing pwCF and chronic *P. aeruginosa* infection who were treated with either an anti-pseudomonal 3-phage cocktail or placebo in addition to inhaled antibiotics. Though the trial was too small to assess efficacy, participants who received inhaled phages had a reduced bacterial burden of *P. aeruginosa* as well as an increase in alpha-diversity observed through 16S metagenomic analysis of their sputum microbiomes (56). Chronic infections with multidrug-resistant pathogens are a significant challenge in this patient population, and these data suggest that phage therapy may prove to be an important adjunct to existing treatment modalities for pwCF in particular.

### Chronic skin ulcers

The treatment of chronic skin ulcerations is another disease state where phage therapy shows promise. Similar to the clinical entities above, chronic ulcers would be amenable to direct, high MOI phage contact via topical preparations, and mechanical debridement is routinely used to reduce the bioburden of colonizing and infecting pathogens from the ulcer surface. In addition to multiple case studies and case series describing the use of phage therapy for chronic skin ulcerations (Table S2), three early-phase clinical trials assessed the safety of phage therapy in the management of chronic wounds caused by either venous stasis or diabetes mellitus (57–59). All three studies reported excellent safety profiles for the phages used. In a trial that enrolled patients infected with bacterial pathogens susceptible to the applied phages, the authors observed reduced microbial burdens and higher rates of key wound closure milestones during follow-up in the phage treatment group compared to those who were not treated with phage (58). While none of these trials were designed to assess efficacy, results suggested possible benefits, and there was no evidence of impaired wound healing with phage treatment.

## DISEASE STATES WHERE CLINICAL EVIDENCE IS LESS CERTAIN REGARDING PHAGE EFFICACY

Phage therapy has been used for a wide variety of clinical indications, and the route of administration, dosage, and duration of therapy have also been highly variable between studies. As such, there are many additional clinical scenarios where the currently available clinical data suggest the promise of phage therapy has yet to be fully realized.

### Burn wounds

The use of phage therapy to treat burn wound infections would seem to be similarly amenable to topical phage treatment as chronic ulcers caused by venous stasis or diabetes. While many case reports and case series report high rates of improvement (23–26), two clinical trials assessing phage therapy for burn wounds performed to date have not shown positive results (21, 22). However, both trials had methodologic issues that may have blunted their efficacy. In reference 21, *P. aeruginosa* and *S. aureus* colonized burn wounds were split into two regions, and only one section was treated with phage therapy while continuing standard-of-care in the neighboring region. Researchers saw no reduction in *P. aeruginosa* or *S. aureus* burden in the areas treated with topical phages as compared to control sites, but the patients spent an extended period of time between study enrollment and phage administration receiving topical treatment with antimicrobial properties across the entire wound surface. It is, therefore, possible that the overall bacterial burden may have been reduced during this time, blunting the effects of phage (21). Additionally, many topical burn wound care products have been found to reduce phage activity *in vitro*, which may have also decreased the effectiveness of phage therapy in this trial (60). Separately, the PhagoBurn trial showed that phage treatment could reduce *P. aeruginosa* colonization of burn wounds; however, the effect was much slower than the standard of care consisting of topical silver sulfadiazine (22). Importantly, the phage preparation used in this trial suffered a dramatic loss in activity prior to application, and bacterial susceptibility was not confirmed before phage application. *Ad hoc* testing showed that wounds infected with more susceptible bacterial populations appeared to display better bacterial clearance compared to wounds containing isolates that were less susceptible at baseline (22), suggesting that the phage susceptibility of colonizing bacteria might have influenced the magnitude of the effect that could have been detected in the trial.

### Acute diarrheal illness

Even in its earliest days, phage therapy for acute bacterial diarrheal illness was not consistently efficacious (61), and a 1970s trial that assessed phage therapy for the acute management of cholera showed that phage was not superior to oral rehydration solution and was inferior to tetracyclines at reducing stool volume and duration of diarrhea (62). In more modern literature, a 2016 trial compared the efficacy of two different enteric-targeting (*E. coli* and *Proteus* spp.) phage cocktails to placebo for the treatment of acute non-viral diarrheal illness in children, and phage therapy also failed to show improvement in diarrheal symptoms compared to placebo (63). It is possible that there was poor activity against the infecting pathogen(s), as neither trial assessed phage susceptibility before phage administration. Additionally, the local MOI of phage preparations in the intestines may be quite variable. Several studies in humans have shown that high doses are necessary for orally administered phage to be reliably detected in stool (64–66). Additionally, the rapid transit time of a diarrheal state coupled with the high density of bacterial flora that could serve as off-target, low-affinity phage “sponges,” may make meaningful interactions between the administered phage particles and their target pathogens difficult. The second phase of a phase 1/2a trial assessing the efficacy of a cocktail of phages active against *Shigella* spp. (ShigActive) is currently pending (NCT05182749), and further studies are needed to determine how best to use phage to manage such illnesses (66).

### Cardiac implantable device-associated bacteremia

Cardiac implantable devices such as pacemakers, defibrillators, and LVADs play an important role in the management of heart failure and other serious heart conditions. Infections of these devices carry high mortality rates and can be challenging to manage. Definitive cure usually requires device extraction, but these procedures carry their own morbidity and mortality risks and may not be feasible in all cases. As such, there has been significant interest in using phage therapy to improve the management of these difficult infections. LVADs and other endovascular devices are in direct contact with the vasculature, and it would be anticipated that systemic phage administration should result in adequate phage-pathogen interactions. Many phages have also been shown to disrupt biofilms through *in vitro* and *in vivo* studies (67, 68) and that property would be anticipated to result in increased likelihood of clinical cure as biofilm formation plays a significant role in many of these hardware-associated infections.

However, in the setting of cardiac implantable device-related infections where the infected device itself cannot be removed or debrided, it appears that phage therapy may be limited in its ability to generate a lasting microbiologic cure. No clinical trials exist assessing the use of phage therapy for these infections. Compassionate use cases present a mixed picture, with a few published examples of prolonged infection resolution, but other studies describe treatment failure or eventual recurrence (69–72). A 2024 review of the case report literature noted an overall rate of clinical improvement of 62% (21/34 patients) when patients with vascular graft or cardiac implantable device-related infections were treated with phage therapy in addition to systemic antibiotics (73). When outcomes were analyzed by whether the patient had concomitant surgery; however, the rates of improvement were quite different between the groups: 83% (15/18 patients) in those who underwent surgery at the time of phage therapy but only 38% (6/16 patients) in those who did not (73). This discrepancy could be because parts of unremovable indwelling cardiac devices may become epithelialized over time, possibly limiting phage exposure. Alternatively, the local concentration of phage at the infection site might be limited by phage pharmacokinetics in the blood, due to their rapid clearance by the reticuloendothelial system, or potentially via interfering substances such as serum proteins.

While lasting microbiologic cure after phage therapy in these infections may not be consistently achievable, there may be some role for phage therapy in the management of these otherwise non-intervenable, chronic implantable device-associated infections. For example, phages could provide additional treatment options if multidrug resistance arises during antibiotic therapy. Phages could also be used in an adjunctive manner with antibiotics to help clear recalcitrant bacteremia or prolong time to recurrence (74), decrease bacterial burden in LVAD driveline infections to reduce the development of bacteremia (71, 75), and/or direct evolution of the infecting pathogen toward beneficial trade-offs like reversion to antibiotic-susceptible or avirulent phenotypes (76). However, resurgence of blood culture positivity (i.e., breakthrough bacteremia) with the infecting pathogen after initiating phage therapy is a potential safety-related concern that needs to be addressed. A prior case series noted breakthrough bacteremia events occurring during phage therapy in patients treated with *P. aeruginosa* LVAD-associated infections (70). While unpublished, this phenomenon has also been noted in a similar patient population at our phage center (Pittsburgh Phage Program). It is hypothesized that these events arise due to breakdown of mature biofilms caused by phage predation, but it is not clear why breakthrough bacteremia has been reported in only a subset of patients. It is also unclear if these transient events pose an additional risk of complications from septic thromboemboli.

## WHAT CAN WE AS A SCIENTIFIC COMMUNITY DO TO IMPROVE PHAGE THERAPY?

Phage therapy is challenging the way we traditionally study and develop new anti-infective treatments. As such, there are large gaps in knowledge that currently prevent phages from realizing their full potential. Here, we highlight research areas where critical gaps in our understanding of phage therapy and phage biology remain.

### Scenarios with limited data on clinical phage use

The benefit of phage therapy for acute infections when patients are severely ill with multi-organ failure is unclear. Most trials of phage therapy to treat clinical infections did not include critically ill patients, and those that did tended to exclude patients at high risk of imminent death (53, 77). Only a few case reports describe successful treatment of severely ill patients (78–80). This is likely due to several reasons. First, finding a phage that is active against the infecting isolate and manufacturing a potent, safe, and usable product takes time, often several weeks to months. Manufacturers must meet a variety of regulatory benchmarks before a phage product is deemed acceptable for clinical use in the United States. In a case series from Green et al., the time from a phage therapy compassionate use request to when the therapy was clinically available was on the order of months (81). Very ill patients may simply not have enough time to wait, or they may be too ill to benefit from phage therapy by the time that an active phage can be identified and administered. Whether phages can be employed efficiently to generate positive clinical effects in this patient population will require more clinical experience.

Another use with limited clinical data is deploying phages as a non-chemical means to reduce the burden of multidrug-resistant pathogens at colonized sites, such as the skin or gastrointestinal (GI) tracts. With their potential for exquisite specificity, phage-based decolonizing agents could be designed to deplete only harmful strains, leaving protective commensals intact and avoiding harsh chemicals that can disrupt the skin barrier. An engineered, broad-spectrum *E. coli* phage cocktail was designed as a GI decolonizing agent for patients undergoing stem cell transplantation, with a goal to reduce resistant *E. coli* infections that can arise during cancer treatment (82). A recent phase 1 trial confirmed that it was well tolerated, and oral administration resulted in detectable active phage particles in the stool (83). In addition, there are two trials registered on the ClinicalTrials.gov online database addressing decolonization of resistant organisms via phage or phage-derived products. One is assessing the use of a phage lysin to eradicate *S. aureus* from the anterior nares (NCT06290557), and the other proposes using a phage cocktail to reduce vancomycin-resistant enterococci from the gastrointestinal tract (NCT05715619). At least two case reports detail the use of phages to successfully decolonize the GI tract and other body sites of multidrug-resistant pathogens (84, 85); however, with limited clinical data, the widespread feasibility of this approach remains uncertain.

### Phage pharmacokinetics and pharmacodynamics

There continue to be many unknowns regarding how phages interact with the human body during treatment. Uncertainty remains about the functional *in vivo* access of phages to their bacterial targets at the site of infection, including the ability of phages to penetrate or translocate through mucosal surfaces and within body tissues as well as the role of non-productive, adsorptive interactions between phages and plasma proteins or cells (86).

These gaps in knowledge hinder the development of clear standards for phage dose ranges and optimal routes of administration. Additionally, similar to how antibiotic minimum inhibitory concentrations are used to choose appropriate antibiotics, we need to know how *in vitro* phage susceptibility testing correlates with *in vivo* clinical efficacy. This information will help us understand how to dose phage products to achieve appropriate *in vivo* phage exposures that reproducibly generate a clinical response and limit side effects. Clinical trial data are growing, especially for infections of the respiratory tract, urinary tract, and skin, and hopefully these studies will add to our knowledge regarding the pharmacologic parameters necessary for effective phage therapy.

Phage therapy is also expanding the limits of current models used to describe antimicrobial pharmacokinetics and pharmacodynamics (PK/PD), which were originally designed to analyze small-molecule effectors (87). However, phages are self-replicating biologics and have the potential to evolve *in vivo*. As such, we are only beginning to understand phage PK/PD, and additional *in vitro* and *in vivo* preclinical data are needed to model the best phage dose and optimum route of administration. A lack of standardized phage susceptibility testing further complicates this issue, as does our limited understanding of how phage parameters like burst size and adsorption kinetics impact therapeutic efficacy (87, 88). To answer these questions, faster and more reliable ways of testing phage activity against a wide variety of phage hosts and correlating these parameters with *in vivo* efficacy are needed. Ideally phage susceptibility testing methods should be amenable to real-time interpretation for physicians administering phage therapy. We also need to incorporate rationally designed PK/PD sample collection protocols into phage clinical trials to gain the crucial data needed to better understand phage PK/PD *in vivo*. These efforts would be aided by the development of more sensitive methods for quantifying phage presence in tissues after phage administration.

### Human immune responses to phage therapy

Phages are the most abundant biological entity on earth, making up a significant part of the external environment and a key component of the human microbiome. However, the interplay between phages and the human immune system is another area where additional research is necessary. In immunocompetent mammalian hosts, phage exposure can lead to the activation of antiviral immunity including cytokine secretion, mucosal inflammation, and the production of phage-neutralizing antibodies (89–92). In fact, the *E. coli*-targeting phage phiX174 has been used for decades as a clinical measure of the human immune system’s ability to mount an appropriate T-cell-mediated humoral response (93). However, in clinical practice immune, system-mediated adverse reactions to phage therapy are rare, which may be related to the fact that humans are continually exposed to phages throughout their lifespans (12, 91, 92). In addition, it remains unclear whether anti-phage immune responses arise to all therapeutic phages and routes of administration and if these responses are potentially detrimental to phage treatment efficacy.

Case reports have documented a lack of sustained clinical response to phage therapy that correlated with the development of neutralizing antibodies (74, 94). Others have detected neutralizing antibody responses to therapeutic phages that did not appear to consistently limit treatment effects (95, 96). Finally, there is also the possibility that an antibody response could be beneficial, with some preclinical *in vivo* data suggesting that phage-mediated bacterial lysis could boost the host immune response to a pathogen, generating durable protection from reinfection with similar strains (97). It is also conceivable that all three scenarios may be possible and that they are context-specific and depend on the pathogen, the site of infection, and the patient’s underlying immune status. To aid in better understanding these complex pathogen-host interactions, the field should standardize how serum neutralization is measured and reported and standardized anti-phage immune response testing should be incorporated into phage therapy clinical trials.

### Phage-antibiotic interactions

Another area of inquiry that would greatly benefit phage therapy development is further research into the interplay between phages and small-molecule antibiotics. In the treatment of human immunodeficiency virus (HIV), tuberculosis, and many types of cancer, combination therapies that target multiple, non-redundant cellular pathways are standard of care and are associated with improved therapeutic efficacy (98–100). Similarly, phage therapy could empower infectious disease physicians to apply these same principles to gram-negative and gram-positive bacterial populations causing infections. The mechanisms by which phages and antibiotics target susceptible bacteria and cause bacterial cell death are very different, and combination therapies including both antibiotics and phages have the potential to provide a more robust therapeutic effect. Clinical trial data for such phage-antibiotic combination therapies are currently limited, but in a significant number of published compassionate use protocols, phages were used as adjunctive therapy to the best available standard of care—usually antibiotics. Clinical experience to date suggests that phage-antibiotic combinations can be more effective than monotherapy with either agent alone, and this idea has also been confirmed in several *in vitro* preclinical studies (101, 102). However, there is also the possibility that some phage-antibiotic combinations could be antagonistic (103–105). While phage-antibiotic interactions are known to vary by phage, host, and antibiotic class, we suggest that synergy should not be the ultimate goal of such testing, but rather testing should focus on confirming a lack of antagonism when combining phages with antibiotics. How best to measure these properties across diverse phage-antibiotic combinations is unknown, and standardized methods to test these interactions in real-time are of utmost importance.

### Phage engineering

Many areas of phage research and phage therapy have the potential to be affected by the next decade’s advancements in phage engineering technology. Our knowledge of phage biology is steadily increasing as more phages are isolated, characterized, and included in preclinical and clinical studies. Coupled with rapid and accurate modern genome editing technologies and artificial intelligence, the phage therapy field is poised to leverage this ever-expanding trove of information toward the optimization of phages for clinical use via the modification of naturally occurring phages and the generation of novel phage genomes. In the near future, researchers will likely be able to readily and rationally modify phages to improve their host range, blunt immune clearance, and/or deliver genetic or nanoparticle payloads to improve treatment outcomes (106). Additionally, cell-free manufacturing techniques can address the difficulty of isolating therapeutic phages from endogenous bacterial toxins and byproducts (107). This topic is vast and exciting, and readers are directed to recent reviews that assess current and emerging phage engineering technologies (106–109).

## HOW CAN INDIVIDUAL RESEARCH PROGRAMS HELP ADDRESS KNOWLEDGE GAPS REGARDING PHAGE THERAPY?

Basic science and pre-clinical research focusing on the gaps outlined above is essential to improve our understanding of phage therapy and accelerate its clinical application. The recent rise in the number of clinical trials focused on phage therapy is encouraging. However, the field can be accelerated by leveraging the experience and knowledge gained through compassionate use protocols to inform phage therapy development. Dozens of patients receive compassionate use phage therapy each year, and standardizing both treatment and monitoring strategies can allow researchers to aggregate our collective experiences and provide clinical data that can be comparable to data gained through clinical trials. Below, we describe our approach at the Pittsburgh Phage Program (P3) as an example of how to standardize and maximize data generation during single-patient and compassionate use phage therapy protocols.

Over the past 8 years, the P3 has treated over 75 patients with phage therapy through compassionate use protocols. These protocols are designed for clinical use and not for research, but to maximize our learning for improving treatment of current and future patients, we developed an Institutional Review Board-approved, standardized phage biorepository. Treatment with phage therapy is not contingent on enrollment in the biorepository, but all patients treated with phage therapy in our hospital system are offered the opportunity to participate. If a patient consents to participate, we collect and store remnant and clinical biospecimens to formally measure phage PK/PD, microbiologic responses, development of phage resistance, and serum antibody responses. To date, we have had 100% participation with only a single patient later withdrawing while continuing to receive phage therapy. Standardizing such approaches across centers providing phage therapy would bring forth new insights and allow for even greater depths of data collection.

## HOW SHOULD WE, AS A COMMUNITY OF SCIENTISTS AND PHYSICIANS, WORK TOGETHER TO IMPROVE PHAGE THERAPY?

It will take a coordinated effort at the national level to realize the full potential of phage therapy. This type of approach has been long-standing at both the Eliava Institute in Georgia and the Phage Therapy Unit in Poland, where both centers have treated hundreds to thousands of patients with phage therapy (110, 111). More recently, other nations are also implementing this approach. Phage Australia has developed the STAMP trial protocol, which has already treated more than 20 patients (112). Since 2018, a Belgian phage therapy consortium has been using compounded personalized phage preparations and accompanying protocols to offer coordinated treatment to over 100 patients both nationally and internationally (113, 114). Phage Canada was formally established in 2023 to facilitate phage therapy in the Canadian provinces (https://www.phagecanada.ca), and Phage UK has also been established to provide a national network to facilitate phage therapy use and research (https://phageuk.org/). In addition, a large, coordinated phage treatment group has also arisen in France (the PHAGEinLYON Clinic), focusing on phage therapy for bone and joint infections (115).

In the United States, there have been recent efforts to establish both phage centers and a coordinated clinical and regulatory network through the National Institutes of Health (NIH) and ASM. The NIH has established centers supporting translational phage research efforts aimed at developing preclinical assays, tools, and models targeting ESKAPE pathogens with recently awarded P01 research program grants. ASM Health recently announced the Phage Therapy Coordination Network (PTCN) initiative (https://asm.org/about-asm/asm-strategic-roadmap/asm-health/phage-therapy-coordination-network), with the goal of supporting clinical research through centralization and standardization of data from compassionate use treatment protocols and the coordination of large-scale, multi-center clinical trials. The PTCN also aims to coordinate scientists, clinicians, and regulatory experts to develop best-practices guidance for both preclinical and clinical phage research. In addition, foundations are providing critical translational research support. For example, the Cystic Fibrosis Foundation has invested heavily in clinical phage therapy research for multidrug-resistant pathogens including two active clinical trials: an observational trial for non-tuberculosis mycobacterial infections (NCT06262282) and an interventional trial for patients colonized with *Achromobacter* species (NCT07275905).

Phage therapy is still in its infancy compared to traditional antimicrobials. Coordinated national efforts focused on addressing key knowledge gaps are crucial, as many current roadblocks to phage therapy development stem from a lack of standardization. Standardization of methods does not mean stagnation or reduction in innovation. In fact, we argue the opposite. Until we define gold-standard assays, similar to the MIC for small-molecule antibiotics, we lack benchmarks by which to evaluate both current therapies and the phage technologies of the future.

## FINAL THOUGHTS

To date, thousands of people have been safely treated with phage therapy worldwide, yet many unanswered questions remain in the field. As we face the possibility of entering a post-antibiotic era, the potential for phage therapy to improve human health is clear. While this field of medicine is primed for paradigm-shifting research in the next decade, it will take the coordinated efforts of researchers and clinicians to harness these insights and take phage therapy into its next century.

## SUPPLEMENTAL MATERIAL

Table S1 mbio.03254-25-s0001.xlsx Data abstraction from 28 phage therapy clinical trials discussed in the text.

Table S2 mbio.03254-25-s0002.xlsx Case reports and case series describing the clinical use of phages for therapy.

ASM does not own the copyrights to Supplemental Material that may be linked to, or accessed through, an article. The authors have granted ASM a non-exclusive, world-wide license to publish the Supplemental Material files. Please contact the corresponding author directly for reuse.
